# A Summertime Stupor

**DOI:** 10.7759/cureus.8242

**Published:** 2020-05-22

**Authors:** Kevin Mauerman, Samuel Durojaye, Gurusaravanan Kutti Sridharan

**Affiliations:** 1 Internal Medicine, University of Arizona College of Medicine, Tucson, USA; 2 Neurology, University of Arizona, Tucson, USA; 3 Internal Medicine, University of Arizona, Tucson, USA; 4 Internal Medicine, Banner University Medical Center - Tucson, Main Campus, Tucson, USA

**Keywords:** hypothermia, encephalopathy, thiamine, wernicke, malnutrition, osborne, pancreatitis waves, opthalmoplegia, ataxia

## Abstract

Most commonly seen with alcohol use disorder in the developed world, Wernicke’s encephalopathy (WE), a disorder caused by thiamine deficiency can be readily missed in the setting of other predisposing conditions such as malnutrition, the most common cause worldwide. We present a case of a 21-year-old female with sudden progressive decline in her level of alertness and mentation along with severe hypothermia who had clinical features and imaging findings consistent with WE in the setting of pancreatitis and chronic gastritis. WE should be suspected in all patients who present with altered mental status (AMS) and who are at an increased risk of malnutrition despite a normal or high body mass index, so that treatment with thiamine may be initiated and further neurologic sequelae can be averted.

## Introduction

Wernicke’s encephalopathy (WE) is a reversible neuropsychiatric disorder caused by thiamine deficiency. The classic triad of presenting symptoms include ataxia, ophthalmoplegia, and confusion; though other rarer presenting signs can include hypothermia, hypotension, and coma [1]. Classically associated with alcohol use disorder in the Western world, it is easily and often overlooked in nonalcoholic patients. Conditions such as hyperemesis gravidarum, starvation, AIDS, eating disorders, and various gastrointestinal conditions have been reported to cause thiamine deficiency and precipitate WE [2-5]. Prompt treatment with thiamine is critical and often curative for these patients especially in the absence of alcohol use disorder [6-7]. Below, we discuss a case where intractable vomiting and abdominal pain in a young female patient with pancreatitis and gastritis led to WE with a rather atypical and sinister presentation.

## Case presentation

A 21-year-old female with a past medical history of questionable phenylketonuria (PKU) not adherent to PKU diet, obesity, and gastritis presented to our facility’s ED with altered mental status (AMS). One month prior to admission, she was diagnosed with gastritis via upper gastrointestinal (GI) endoscopy at a different hospital for symptoms of epigastric pain and nausea/vomiting (N/V). She was prescribed omeprazole which she stopped taking as it provided no symptom relief. Two weeks prior to admission - during her initial visit to our facility, she presented to the ED with decreased oral intake, epigastric pain, N/V, dizziness, and shortness of breath which had been progressing since being diagnosed with gastritis. She was treated symptomatically in the ED and was discharged home in stable condition to follow up with a gastroenterologist and primary care physician.

It was upon her second presentation to our facility’s ED that she was found to have AMS. Per history obtained from relatives at the time, she had locked herself in her bedroom two days prior to presentation and was found on the morning of admission laying on the floor next to her bed. In addition, the family stated that her symptoms of abdominal pain and N/V had persisted over the last two months and that over the last two weeks, her N/V worsened so much so she could no longer tolerate food without vomiting. Simultaneously, she was also experiencing progressive ataxia, blurry vision, and increasing confusion to the point where she could no longer engage in meaningful conversations.

Upon evaluation in ED, she had a Glasgow coma scale score of 10 (eye response-4, verbal response-1, motor response-5). In addition, she was notably hypothermic with a core temperature of 26.7°C after rewarming. Vitals were: blood pressure 114/55 mmHg, heart rate 54/min, respiratory rate 15/min, temporal temperature 26.4°C (core temperature not initially detected by the thermometer). A physical exam by ED physician revealed slowed mentation without appropriate response, cold skin, pallor, and bradycardia.

Pertinent workup and significant labs include (see Table 1):

**Table 1:** Laboratory values. BUN: blood urea nitrogen, Cr: creatinine, T. Bili: total bilirubin, AST: aspartate aminotransferase, ALT: alanine aminotransferase, CK: creatinine kinase, β-HBA: beta-hydroxybutyric acid

Lab	Value (hospital lab reference range)
Platelet	550,000 /mm^3^ (130,000-450,000)
Sodium	125 mmol/L (135-145)
Potassium	1.9 mmol/L (3.6-5.3)
Chloride	67 mmol/L (96-100)
BUN	57 mg/dL (8-25)
Cr	2.19 mg/dL (0.6-1.4)
Glucose	436 mg/dL (65-99)
Anion gap	27
T. Bili	2.0 mg/dL (0.2-1.3)
AST	62 IU/L (10-41)
ALT	101 IU/L (10-46)
Lipase	521 IU/L (8-75)
CK	1628 IU/L (21-215)
Lactic acid	6.9 mmol/L (0.5-2.0)
β-HBA	32.8 mg/dL (less than 2.8)

**Figure 1:**
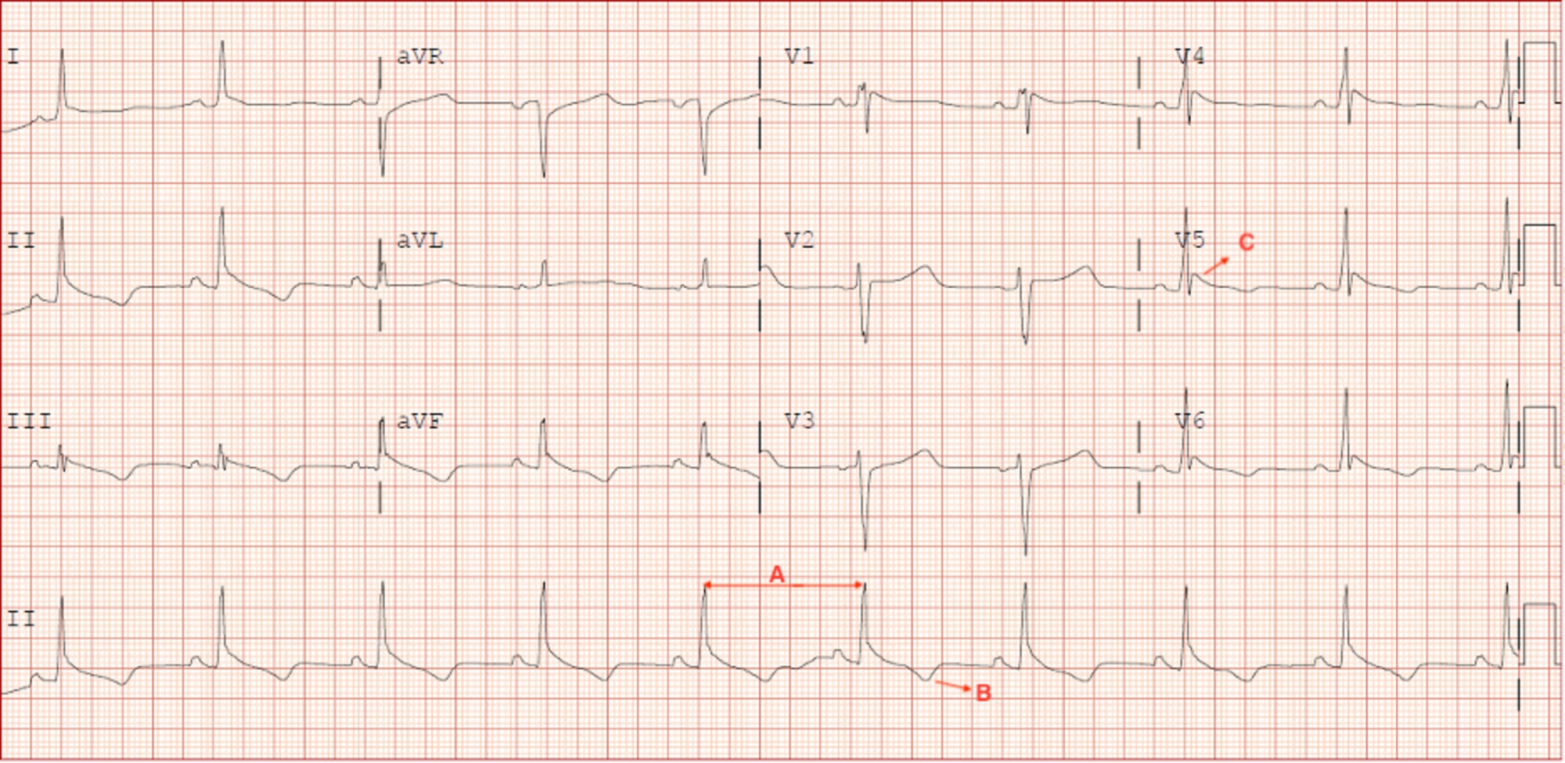
12 Lead EKG. Her EKG shows sinus bradycardia (A), T wave inversions (B), and Osborn wave (C) in  leads V4-V6 EKG, electrocardiogram

Her alkaline phosphatase (ALP) was within normal limits as were her thyroid studies and her arterial blood gas (ABG) co-oximetry. Her pregnancy screen was negative. Urine drug screen was positive for cannabinoids and urinalysis suggestive of urinary tract infection (UTI), mild proteinuria, and hematuria. Her serum alcohol level was zero and triglyceride level within normal limits (WNL). See Figure 1 below.

**Figure 2:**
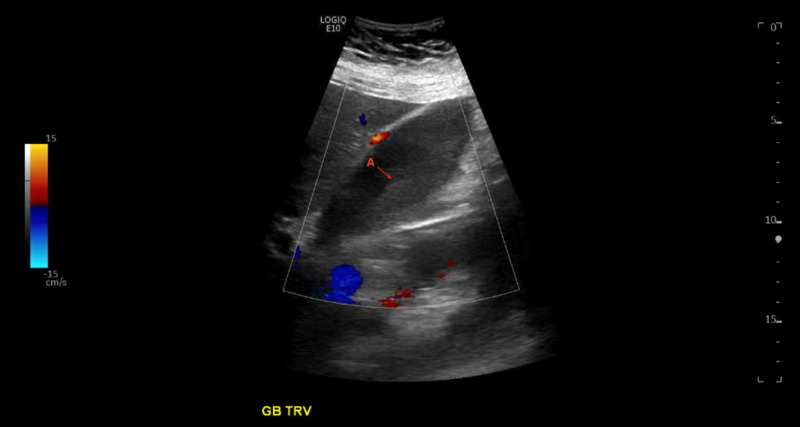
Gallbladder ultrasound. Ultrasound of the gallbladder showing biliary sludge (A)

Her electrocardiogram (EKG) showed sinus bradycardia with Osborn wave and a CT scan of the abdomen and pelvis showed findings of mild pancreatitis. Biliary sludge was seen on abdominal ultrasound without biliary ductal dilation or gallstones and no signs of hepatobiliary inflammation. See Figure 2.

**Figure 3:**
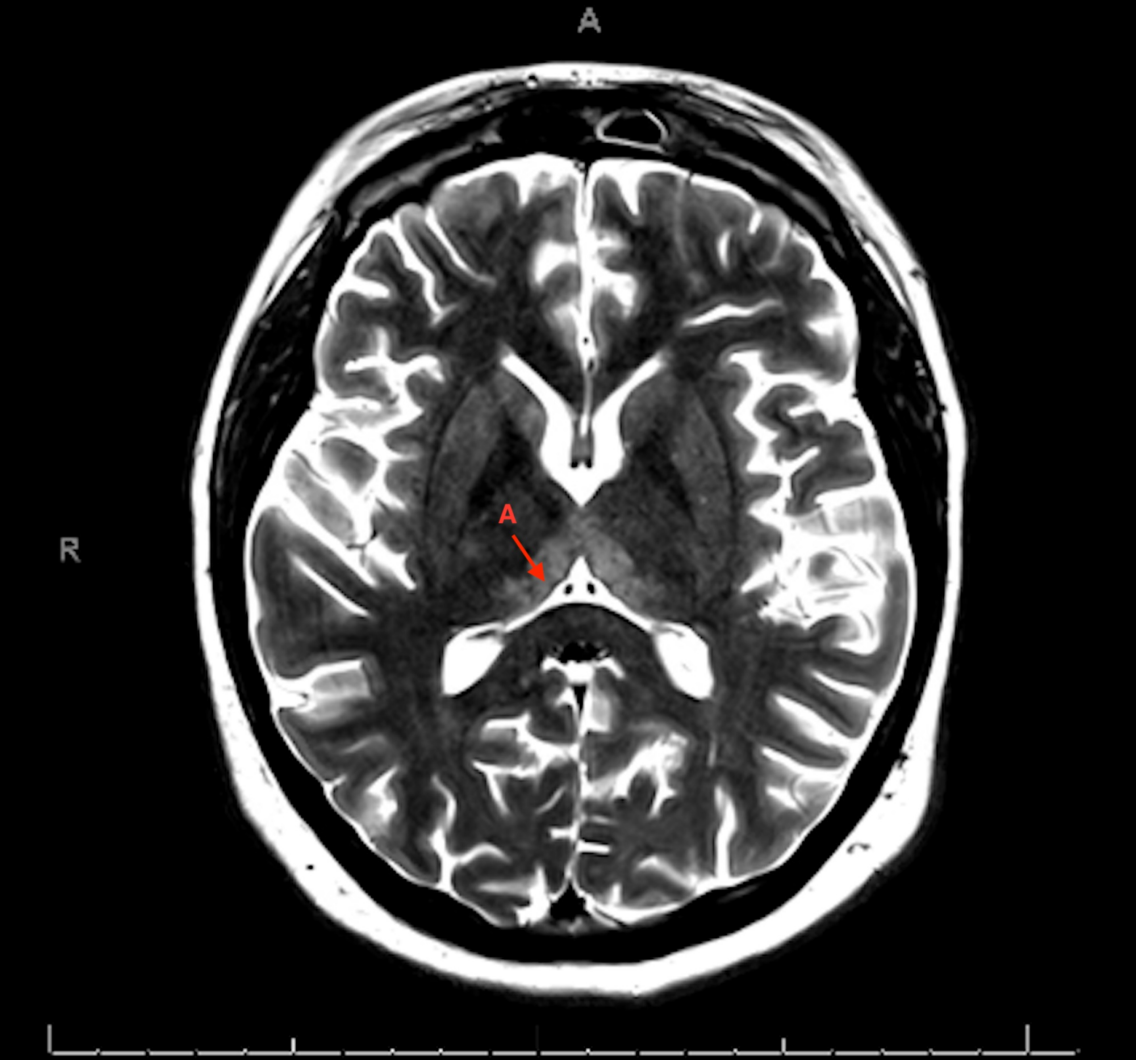
MRI of the brain. T2 sequence showing symmetric bilateral hyperintensities in the dorsomedial thalami (A) marked with red arrows

**Figure 4:**
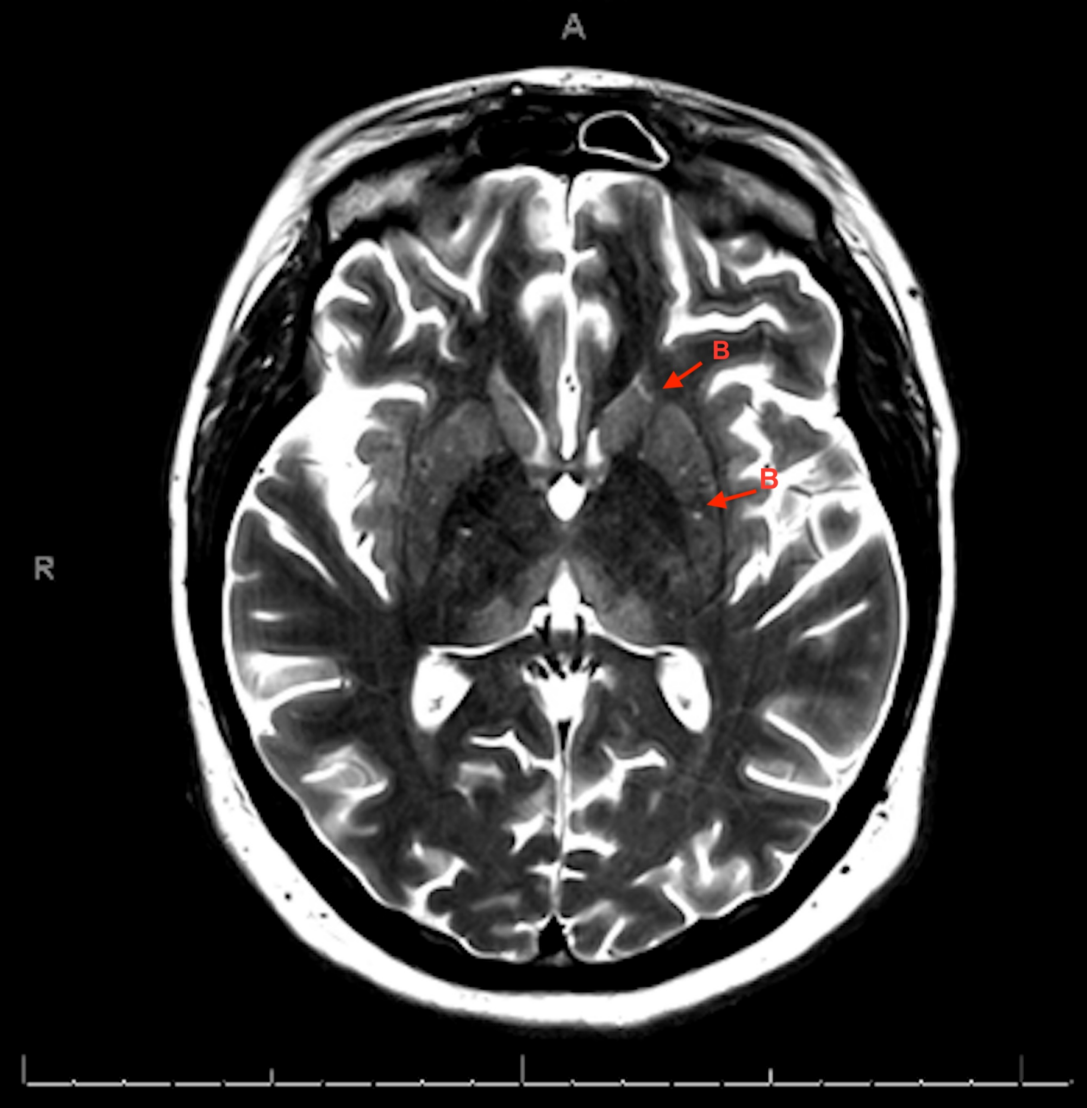
MRI of the brain. T2 sequence showing symmetric bilateral hyperintensities in the basal ganglia structures (B) marked with red arrows

She was admitted to the pediatric ICU (given her history of PKU) where she was treated with appropriate intravenous fluid resuscitation and then initially maintained on high rate of maintenance fluid given intravascular depletion, rhabdomyolysis, and acute kidney injury. She was actively rewarmed to normothermic body temperatures, and she was placed on empiric antibiotics for her presumed UTI. She was also started on folic acid and thiamine for presumed malnutrition while her electrolyte abnormalities were rapidly addressed. Her mental status gradually improved over the next several days. After stabilization, she was transferred to our adult medicine service from the pediatric ICU. At the time of transfer, physical exam revealed left abducens nerve palsy (ophthalmoplegia), facial myoclonus, ataxia when walking as well as with finger to nose testing, and slurred speech. Subsequent MRI of her brain revealed symmetrically increased T2 signaling in her medial dorsal thalami and basal ganglia consistent with WE following which her dose of thiamine was increased. See Figures 3-4

Our patient was found to have classic triad of symptoms along with other rare manifestations of this syndrome and supporting imaging findings. She was diagnosed with WE secondary to prolonged malnutrition in the setting of intractable vomiting caused by gastritis and pancreatitis. Following initiation of a higher dose of intravenous thiamine: 500 mg TID, she had a rapid recovery. Her encephalopathy resolved dramatically over the subsequent 48 hours, as did her gait ataxia, slurred speech, and blurry vision. She also had complete resolution of her ophthalmoplegia. She still had some residual, albeit much improved, myoclonus at the time of discharge though this also showed continuous improvement during her inpatient stay. She was discharged to a rehabilitation facility for physical therapy on high dose thiamine and a multivitamin supplement with follow-ups arranged with PCP and Neurology. PKU was later confirmed upon discharge based on lab findings.

## Discussion

Pathophysiology of Wernicke’s encephalopathy

Wernicke’s encephalopathy occurs as a result of thiamine deficiency resulting in intracellular depletion of thiamine in brain cells which led to increased metabolic demand and subsequent energy deficit. This deficit results in increased oxidative stress focally as in local acidosis with increased production of glutamate and nitric oxide resulting in eventual cell death [7].

Thiamine is a water-soluble vitamin. The daily thiamine requirement for a healthy adult is about 1-2 mg per day. It is primarily absorbed in the small intestine and body stores are between 25 and 50 mg and is primarily in the liver [7-8]. It has a very short half-life as such a continuous supply is required to keep up with storage and daily requirement. Based on daily requirements, it can be postulated that body stores can be depleted in four to six weeks [5-6, 8]. The active form of thiamine is thiamine diphosphate (TDP) and is involved in the metabolism of glucose, amino acids, and lipids [8].

Manifestation: history, symptoms, and medical imaging

Wernicke’s encephalopathy is a neuropsychiatric emergency that carries up to 20% mortality and high morbidity [6]. It results from an acute deficiency of thiamine and is associated with the classic triad of ataxia, mental status changes, and ophthalmoplegia. The triad is present in a mere 10%-16% of WE, which is one of the reasons why the diagnosis is readily missed [5-6]. AMS is by far the most common presentation followed by ocular signs and then gait disturbances. Other rarer but known presenting symptoms such as: hypothermia, hypotension, and coma are worth noting [1]. A sequela which is seen in chronic thiamine deficiency is Korsakoff psychosis where there is irreversible, persistent, and severe impairment of working memory [6]. Our patient did present with acute and rapidly worsening mental status change, blurry vision with dysconjugate eye movement, hypothermia and on subsequent exams, an ataxic gait.

Imaging findings that characterize WE are best seen on MRI as opposed to CT imaging as the latter is less sensitive in identifying these changes. The MRI typically shows symmetric alterations in the thalami, mamillary body, tectal plate, and periaqueductal gray. Less typical lesions are symmetric changes in the cerebellum, vermis of the cerebellum, and cranial nerve nuclei among other areas [5]. Changes in the medial thalami and periventricular regions of the third ventricle are said to be the most common areas of changes seen on the MRI in acute WE [5]. 

It is likely that in our patient, WE was precipitated by acute thiamine deficiency from malnutrition as a result of frequent vomiting and reduced oral intake secondary to her pancreatitis and chronic gastritis that had been ongoing for nearly eight weeks; enough time for her thiamine stores to be depleted resulting in her developing WE. Unfortunately, in WE there is no standard dosing for thiamine supplementation and guidelines vary. However, it is known that prompt intravenous thiamine supplementation in high doses is better than oral route of supplementation as the rate and amount absorbed from the gastrointestinal system does not provide sufficient replacement in WE [6-8]. Thiamine in higher concentrations in serum can readily pass through the blood-brain barrier via passive diffusion. At our facility, we treat with thiamine 500 mg IV TID (three times a day) and then orally with 250 mg daily for five days. Those with a high risk of recurrence as with patients who suffer from alcohol use disorder are placed on 100 mg of thiamine daily thereafter for an extended period.

## Conclusions

Due to the reversibility, ease of treatment, and high fatality rates of WE, it remains paramount for clinicians to have a high suspicion for diagnosing WE in all patients presenting with AMS especially in those not suffering from alcohol use disorder. Caution is to be exercised in obese patients as malnutrition is often underrecognized in this population. Thiamine levels lack sensitivity and specificity for diagnosing active disease and also not readily available. MRI imaging can identify WE in two thirds of patients, however, not required for diagnosis. Administration of high dose intravenous thiamine remains the mainstay of treatment and WE, while a medical emergency, is known to have excellent response to timely treatment.
